# Actin-Cytoskeleton Drives Caveolae Signaling to Mitochondria during Postconditioning

**DOI:** 10.3390/cells12030492

**Published:** 2023-02-02

**Authors:** Francisco Correa, Cristina Enríquez-Cortina, Alejandro Silva-Palacios, Nadia Román-Anguiano, Aurora Gil-Hernández, Marcos Ostolga-Chavarría, Elizabeth Soria-Castro, Sharik Hernández-Rizo, Paola de los Heros, María Chávez-Canales, Cecilia Zazueta

**Affiliations:** 1 Departamento de Biomedicina Cardiovascular, Instituto Nacional de Cardiología Ignacio Chávez, Juan Badiano No. 1, Colonia Sección XVI, Mexico City 14080, Mexico; 2 Área de Medicina Experimental y Traslacional, Departamento de Ciencias de la Salud, Universidad Autónoma Metropolitana-Iztapalapa, Mexico City 14080, Mexico; 3 Unidad de Investigación UNAM-INC, Instituto Nacional de Cardiología Ignacio Chávez and Instituto de Investigaciones Biomédicas, Universidad Nacional Autónoma de México, Mexico City 14080, Mexico

**Keywords:** reperfusion damage, caveolae, mitochondria, postconditioning, actin-cytoskeleton

## Abstract

Caveolae-associated signaling toward mitochondria contributes to the cardioprotective mechanisms against ischemia-reperfusion (I/R) injury induced by ischemic postconditioning. In this work, we evaluated the role that the actin-cytoskeleton network exerts on caveolae-mitochondria communication during postconditioning. Isolated rat hearts subjected to I/R and to postconditioning were treated with latrunculin A, a cytoskeleton disruptor. Cardiac function was compared between these hearts and those exposed only to I/R and to the cardioprotective maneuver. Caveolae and mitochondria structures were determined by electron microscopy and maintenance of the actin-cytoskeleton was evaluated by phalloidin staining. Caveolin-3 and other putative caveolae-conforming proteins were detected by immunoblot analysis. Co-expression of caveolin-3 and actin was evaluated both in lipid raft fractions and in heart tissue from the different groups. Mitochondrial function was assessed by respirometry and correlated with cholesterol levels. Treatment with latrunculin A abolishes the cardioprotective postconditioning effect, inducing morphological and structural changes in cardiac tissue, reducing F-actin staining and diminishing caveolae formation. Latrunculin A administration to post-conditioned hearts decreases the interaction between caveolae-forming proteins, the co-localization of caveolin with actin and inhibits oxygen consumption rates in both subsarcolemmal and interfibrillar mitochondria. We conclude that actin-cytoskeleton drives caveolae signaling to mitochondria during postconditioning, supporting their functional integrity and contributing to cardiac adaption against reperfusion injury.

## 1. Introduction

Caveolae are invaginations of the plasmatic membrane considered as a “lipid raft” subtype, enriched with cholesterol, sphingolipids and specific proteins, such as cavin, pacsin, EHD2 and mainly, with caveolin (cav). This protein participates in several cellular processes, such as vesicular and cholesterol transport, calcium homeostasis and space-temporal regulation of signal transduction [1]. Of the three caveolin isoforms, cav-3 is expressed in skeletal and cardiac muscle, and to a lesser extent in smooth muscle cells; cav-1 is expressed in adipocytes, endothelial and smooth muscle cells, whereas cav-2 is found in other cellular types [2]. All of them possess scaffold domains for the anchorage of different proteins [3], including members of the reperfusion injury salvage kinase (RISK) pathway [4], that phosphorylate and regulate the activity of intracellular targets, reducing cardiac reperfusion damage [5]. A well-known example is the MEK1/2-ERK1/2-GSK3β (mitogen-activated protein kinase/extracellular signal-regulated kinase 1/2-glycogen synthase kinase 3 beta) cascade of the RISK family, activated after GPCR (protein-coupled receptor) stimulation, that interacts with mitochondrial membranes, regulating the activities of the permeability transition pore (mPTP) [6] and the ATP-dependent mitochondrial K+ channels (mitoKATP) [7]. In this sense, we and others have shown that plasmatic structural modules enriched with cav-3, contain cardioprotective kinases [8] that interact with mitochondria [9], reinforcing the idea that maintenance of the mitochondrial function is paramount for cardio-protection, since loss of their integrity is considered the “non-return” point for cardiac cells’ fate after reperfusion [10]. Accordingly, preserved mitochondrial function is a consequence of maneuvers or strategies that prevent cardiac reperfusion damage.

The impact of the associations between the plasmatic membrane and intracellular compartments, as well as their contribution to physical and signaling processes, is a buoyant field in cardiology research. Sub-plasmalemma microdomains proximal to the mitochondria interact physically with them, producing post-translational modifications in mitochondria that regulate their function [9,11]. The demonstration that caveolae constitute platforms that deliver cardioprotective signaling to mitochondria and preserve their function has been provided by our group [12]; however, the involved driving mechanisms remain controversial.

In this work, we aim to determine if the actin-cytoskeleton, that participates in maintaining the structural organization of eukaryotic cells [13], might regulate the cardioprotective signaling transmission of caveolae to mitochondria. In this regard, it is known that actin-cytoskeleton disruption interrupts the GPCR-associated signaling [14], and diminishes caveolin phosphorylation associated with adenylate cyclase inactivation in cardiomyocytes [15]. Furthermore, some cytoskeleton proteins have binding sites to caveolin [4] and the caveolar protein pacsin 2, has reported to interact directly with actin [16]. Therefore, in this paper we test the hypothesis that cytoskeleton disruption destabilizes caveolae, mitigating the cardioprotective effect of postconditioning (PostC) associated with a loss of mitochondrial function.

## 2. Materials and Methods

This investigation was conducted following the Guide for the Care and Use of Laboratory Animals, published by the United States National Institutes of Health (US-NIH) and approved by the research committee of the National Institute of Cardiology I. Ch (INC-13806). Experimental work followed the guidelines of the Mexican Official guide for the use and care of laboratory animals (NOM-062-ZOO-1999) and for the disposal of biological residues (NOM-087-SEMARNAT-SSA1-2002). Most reagents were purchased from Sigma Aldrich (Sant Louis, MO, USA). The chemiluminescent detection system was obtained from Millipore (Bedford, MA, USA). Phalloidin-iFluor 405 reagent (ab176752), phalloidin-iFluor 647 reagent (abcam176752), latrunculin A (ab144290), anti-caveolin-3 caveolae marker (ab2912) and anti-PTRF (cavin-1, ab-76919) were purchased to Abcam (Cambridge, UK); whereas anti-LDL-R (sc-18823), anti-actin mAb (sc-8432), anti-pacsin-3 mAb (sc-373952) and anti-EHD2 mAb (sc-390513) were purchased from Santa Cruz Biotechnology (Dallas, TX, USA). G-Actin/F-actin In Vivo Assay Biochem Kit (Cat. # BK037) was obtained from Cytoskeleton, Inc. (Denver, CO, USA).

### 2.1. Experimental Design

**Figure 1:**
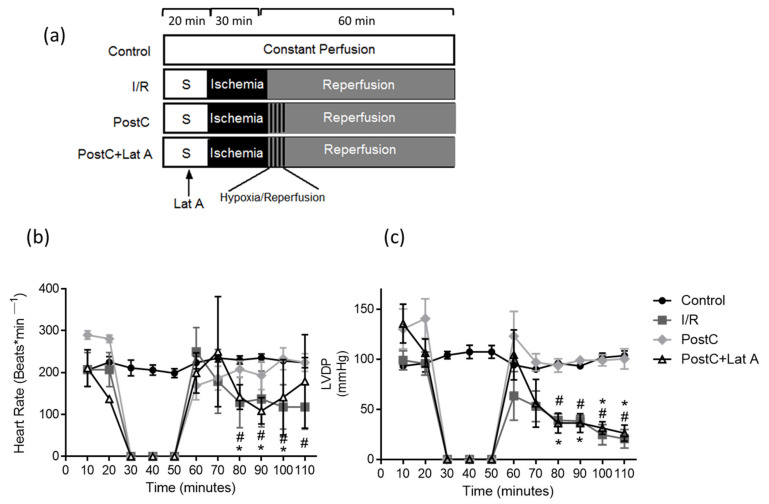
Latrunculin A abolishes cardiac function in post-conditioned hearts. (**a**) Schematic representation of the experimental protocols: hearts perfused for 110 min (Control), hearts subjected to 20 min of stabilization, 30 min of ischemia, and 60 min of reperfusion (I/R), I/R hearts with hypoxic postconditioning (PostC) and PostC hearts treated with 1 μM of latrunculin A before ischemia. (**b**) Heart rate and (**c**) left ventricular diastolic pressure (LVDP) evaluated for 110 min. Values are the mean ± SEM, n = 6–8 different preparations. * *p* < 0.05 vs. PostC; # *p* < 0.05 vs. Control.

Male Wistar rats (250–300 g) were anesthetized with a single dose of sodium pentobarbital (60 mg/kg i.p) and a complete lack of pain response was assessed by determining the pedal withdrawal reflex. The hearts were rapidly excised via midsternal thoracotomy, placed shortly in ice-cold Krebs–Henseleit buffer, and perfused retrogradely via the aorta at a constant flow rate of 12 mL/min with Krebs–Henseleit solution, which was continuously bubbled with 95% O_2_ and 5% CO_2_ at 37 °C. Cardiac performance was measured at the left ventricular end-diastolic pressure (LVEDP) of 10 mmHg using a latex balloon inserted into the left ventricle and connected to a pressure transducer and to the PowerLab System (ADInstruments, Sydney, NSW, Australia). Throughout the experiment, the left ventricular developed pressure (LVDP) and heart rate (HR) were calculated automatically from the pressure trace with the digital acquisition system LabChart 8.1.5 Pro (ADInstruments, Sydney, NSW, Australia). HR is expressed as beat number min^−1^ and the double product (DP) was calculated by multiplying the HR by LVDP [17]. The hearts were perfused for 20 min to reach a steady state and then subjected to the different protocols. The experimental groups were: (1) Control, hearts perfused for an additional 90 min; (2) I/R, hearts subjected to global ischemia for 30 min by turning off the pumping system and 60 min of reperfusion; (3) PostC, hearts subjected to 30 min of ischemia, to postconditioning (5 cycles of 30 s reperfusion and 30 s ischemia) and to 60 min of reperfusion and (4) PostC + Lat A, post-conditioned hearts to which 1 μM of latrunculin A were administrated for 10 min before ischemia (Figure 1a), as reported by Smyth et al. [18]. Cytoskeletal disruption with latrunculin A, might cause changes in the cell structure and signaling, therefore we determined whether such a dose and timing might exert a different effect on the Control and PostC heart function, unraveling a possible link between the cell structure and PostC signaling. Control hearts preserved both the HR and LVDP, whereas PostC showed diminished contractility and higher heart rates at the end of the experiment (Supplementary Figure S1), suggesting that the loss of PostC-conferred cardio-protection has an additional component besides actin damage.

### 2.2. Electron Microscopy

At the end of the experiment, the ventricular tissue was sectioned into pieces of 1 mm of thickness, fixed by immersion in 2.5% glutaraldehyde for 1 h at 4 °C, post-fixed in 1% OsO_4_, dehydrated with increasing concentrations of ethanol and infiltrated with Epon 812 (Electron Microscopy Sciences). Ultrathin sections of 60 nm contrasted with uranyl acetate and lead citrate were examined with a JEM-1011 JEOL Ltd., Tokyo, Japan) at 60 kV to detect the mitochondrial areas and cristae-integrated density. The Multi Measure ROI tool of ImageJ 1.48 software, was used to analyze the digital images obtained at similar magnifications. The total number of caveolae per membrane length identified in both open (structures visibly integrated into the sarcolemma) and closed (as vesicles) configurations were counted and compared between the experimental groups.

### 2.3. Phalloidin Staining and F-Actin/G-Actin Levels

For phalloidin staining, deparaffinized heart sections were incubated with phalloidin-iFluor 647 conjugate for 2 h at room temperature. Fluorescence microscopy images were obtained with a research fluorescence microscope (Olympus, Tokyo, Japan; Leica Microsystems, Wetzlar, Germany) equipped with a digital camera. A cellular fractionation protocol was also used to measure F-actin and G-actin levels in the heart tissue from each group (G-actin/F-actin in vivo assay, cytoskeleton, Cat. #BK037). Briefly, 0.1 mg of frozen heart tissue was treated with 1 mL of warm LAS2 buffer (1 mL of lysis and F-actin stabilization buffer, 10 µL of ATP stock solution (100 mM), 10 µL of 100× protease cocktail inhibitor) and homogenized using a motorized homogenizer. Lysates were incubated at 37 °C for 10 min. Then, a 100 µL volume from each lysate was saved for further analysis. The rest of the sample was centrifuged at 350× *g* in a tabletop microfuge at room temperature for 5 min to pellet the unbroken cells of tissue debris. The obtained supernatants were transferred into clearly labeled ultracentrifuge tubes. Supernatants were centrifuged at 100,000× *g* at 37 °C for 1 h to pellet F-actin and leave G-actin in the supernatant. The obtained supernatants were transferred to fresh tubes designated as supernatant samples. Then, 100 µL of F-actin depolymerization buffer (supplied in the kit) was added to each pellet and incubated on ice for 1 h to allow for actin depolymerization. The supernatants were pipetted up and down several times every 15 min. Then, we added 25 µL of 5X SDS sample buffer to each of the pellets and supernatant samples and mixed well. Samples were tested by SDS-PAGE and western blot analysis.

### 2.4. Immunofluorescence

Tissues were perfused with PFA 4% for immunofluorescent labeling. Frozen OCT-embedded sections were cut to 5 μm and epitope retrieval was performed in citrate buffer (10 mM sodium citrate, pH 6.0). Sections were blocked with donkey antiserum 5% (Jackson ImmunoResearch Laboratories, code 017-000-121) and bovine serum albumin 1% % (Jackson ImmunoResearch Laboratories, code 001-000-162). Primary and secondary antibodies were dissolved in blocking buffer and incubated overnight at 4 °C. Labeling was performed with anti-actin antibody, diluted 1:500 (Abcam, ab20272), anti-caveolin-1 antibody-caveolae marker diluted 1:1000 (Abcam 18199); anti-caveolin-3, diluted 1:1000 (Abcam, ab2912) and with phalloidin-iFluor 405 reagent (Abcam, ab176752), as specified. Fluorescently labeled secondary antibodies (Jackson ImmunoResearch Laboratories) were dissolved in blocking buffer and applied for 2 h at room temperature. As indicated, the cell nuclei were counterstained with DAPI with blue fluorescence. Samples were mounted with Fluoromount mounting media (Thermo-Fisher, 00-4959-52). Images were acquired using a laser-scanning confocal microscope system (LSM 700, Carl Zeiss, Zeiss, Germany).

### 2.5. Caveolin-3 and Actin Segregation in Lipid Rafts

Triton X-100 solubilization and the sucrose gradient centrifugation protocol was performed as follows. Frozen powdered heart tissue (300 μg) from all groups was lysed in 3 mL of MBS (25 mM MES and 150 mM NaCl, pH 6.5) containing 1% Triton X-100 and supplemented with a protease inhibitor mix (Complete™ Protease Inhibitor Cocktail (Roche Molecular Biochemicals). The samples were incubated at 4 °C for 60 min under constant rotation, and the debris was removed by centrifuging at 3000 RPM for 10 min. Then, 1 mL of the homogenate was mixed with an equal volume of 80% (*w*/*v*) sucrose in MBS and placed at the bottom of a centrifuge tube, to be further overlaid with 10 mL of MBS buffer with 35% sucrose and finally with 10 mL of MBS buffer plus 5% sucrose. Following centrifugation at 26,000 RPM in a Beckman SW28Ti rotor for 18 h, 1 mL fractions were collected from the bottom of the gradient [designated fractions number 1 (top) through 11 (bottom)] and concentrated to 0.5 mL for the protein content measurement and western blot analysis.

### 2.6. Western Blot Analysis and Co-Immunoprecipitation

Clarified heart homogenates (2 mg) were mixed with 10 µg of polyclonal anti-cav-3 antibodies, along with nonspecific mouse and rabbit IgGs (5 µg) for 1 h at 4 °C. Then, 20 µL of protein A beads (Santa Cruz Biotechnology) were added for 1 h under constant agitation at a cold temperature. The supernatant with unbound proteins was collected and the beads were washed four times with 1 mL of a buffer containing 50 mM Tris/HCl, 1 mM EGTA, and pH 7.5. Bound proteins were eluted with 1× SDS-PAGE loading buffer containing 2% SDS and subjected to PAGE and western blot.

### 2.7. Subsarcolemmal and Interfibrillar Mitochondrial Isolation

Heart tissue was finely minced and washed in a cold buffer containing 100 mM KCl, 50 mM Tris base, 5 mM MgCl_2_, 1 mM EDTA, 1 mM ATP; pH 7.5 (buffer A). Subsarcolemmal (SSM) and interfibrillar (IFM) subpopulations were obtained as described by García-Niño et al. 2017 [12]. For the SSM isolation, heart tissue was homogenized in buffer A with five strokes of a Teflon pistil in a glass potter. The homogenate was clarified by centrifuging at 750× *g* for 10 min, and then the supernatant spun down for 10 min at 12,000× *g*. The pellet was suspended in 220 mM sucrose, 70 mM mannitol, 10 mM Tris base, 1 mM EDTA, pH 7.4 (buffer B) supplemented with 1.5% albumin fatty acid-free (AFAF) and incubated for 10 min on ice, before further centrifuging at 12,000× *g*, 10 min. The pellet was resuspended in the same buffer without albumin. For IFM isolation, the sediment of the first centrifugation was suspended in buffer A supplemented with 0.250 mg/mL of the protease nagarse and incubated for 5 min on ice. Then, homogenization was performed using a Teflon pistil in a glass potter (five strokes) and the suspension was centrifuged at 750× *g* for 10 min. The supernatant was centrifuged for 10 min at 12,000× *g* and the resulting pellet was suspended in buffer B supplemented with 1.5% albumin AFAF, incubated further for 10 min on ice, and finally, centrifuged at 12,000× *g* for 10 min. The mitochondrial pellet was collected and suspended in a small volume of buffer B. Mitochondria were further purified by 60% percoll gradient ultracentrifugation. Protein concentration was determined by the Lowry method [19].

### 2.8. Cholesterol Measurement in Isolated Mitochondria

For the total cholesterol determination, 100 µL of isolated mitochondria suspension was saponified by using alcoholic KOH in a 60 °C heating block for 30 min. Once the mixture had cooled, 666 µL of hexane and 600 μL of distilled water were added and shaken to ensure complete mixing. Appropriate aliquots of the hexane layer were evaporated overnight and used for cholesterol measurement with O-phthalaldehyde dissolved in acetic acid (0.5 mg/mL). Then, sulfuric acid (300 µL) was added and then read at 550 nm in a spectrophotometer. A curve of cholesterol (0 µg–100 µg) was used as the standard [20].

### 2.9. Mitochondrial Function

O_2_ consumption rate (OCR) was measured in isolated interfibrillar and subsarcolemmal mitochondria with an Agilent Seahorse XFe/XF24 analyzer. Substrates and inhibitors were prepared in 1XMAS buffer supplied by the manufacturer. A 10X concentration of the compounds was added to the corresponding ports to achieve the following final concentrations: ADP (adenosine 5′–diphosphate, 2 mM, 50 μL); oligomycin (2 μM, 55 μL); CCCP [carbonyl cyanide chlorophenylhydrazone, 6 µM, 60 μL]; and 2 μM antimycin-A (65 μL). Isolated mitochondria were diluted to 1 mg/mL with MAS buffer supplemented with free-fatty acid bovine serum albumin 0.2%. Then, 15–20 μg of protein per well were loaded in cold and the plate was centrifuged at 2500× *g* for 15 min at 4 °C. Homogenous adherence to the wells was confirmed after viewing the mitochondria under the microscope. Thereafter and always in cold, 445 μL of 0.5 M malate-glutamate solution was added and incubated for 8 min at 37 °C before placing the plate in the XF24 analyzer.

### 2.10. Statistical Analysis

Data are presented as the mean  ±  SEM. A one-way or two-way repeated measure ANOVA was used to determine the statistical significance among multiple groups’ results, followed by Tukey’s multiple comparisons test. For all statistical analyses, a *p*  <  0.05 was considered to be statistically significant. GraphPad Prism (version 7.0, GraphPad Software, Inc., La Jolla, CA, USA) was used to create artwork and for all statistical tests.

## 3. Results

### 3.1. Changes in the F-Actin Microfilament Cytoskeleton Abolish the Cardioprotection and Reduce the Caveolae Content in PostC Hearts

Heart rate values in the control hearts were around 200 beats/min during the experiments. In the I/R group, the heart rate increased in early reperfusion due to the onset of arrhythmias (249.6 beats/min) and diminished significantly, as compared to the Control (118 ± 53 vs. 223 ± 12 beats/min, *p* ≤ 0.05) at the end of reperfusion. Moreover, recovery of the heart rates in PostC hearts was lost in the presence of latrunculin A at some time points during reperfusion. The left-ventricular developed pressure (LVDP) which was constant in the Control hearts (93.3–107 mmHg) during 110 min of constant perfusion, while LVDP dropped from the first minute until the end of reperfusion in the I/R hearts (63.33–20.66 mmHg). The PostC group maintained LVDP values between 93.33 and 123 mmHg along reperfusion, whereas the PostC + Lat A group showed lower LVDP during reperfusion (56–26 mmHg). The loss of both the heart rate and LVDP in the post-conditioned hearts treated with latrunculin A (PostC + Lat A) was observed (Figure 1b,c), suggesting that the actin-cytoskeleton preservation is required to maintain PostC driven protection.

**Figure 2:**
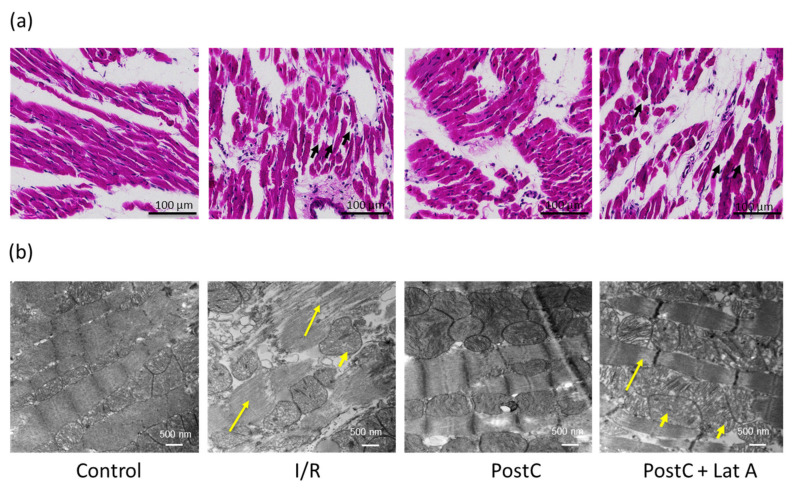
Latrunculin A induces changes in the cardiac ultrastructure of post-conditioned hearts. Representative hematoxylin/eosin staining (**a**) showing cardiac fibers with acidophilic sarcoplasm and oval nuclei in the Control and PostC hearts; disorganized fibers are marked with black short arrows. (**b**) Representative electron microscopy images show fiber rupture (long yellow arrows) and swollen mitochondria (short yellow arrows). The area evaluated for each slice corresponds to the left ventricle in the middle zone. This analysis was performed in a blinded manner, the pathologist who examined the samples has no knowledge of the treatment group.

Morphologic and ultrastructural analyses with eosin/hematoxylin staining and electron microscopy showed longitudinally arranged cardiac fibers, with acidophilic sarcoplasm and central oval nuclei in the Control and PostC hearts; whereas the disorganized sarcomeric structure was observed in hearts subjected to I/R and in the PostC + Lat A group (Figure 2a). We quantify the nuclei number as a measure of viable cardiomyocytes, using H&E stained tissue with the IHC Plugin Fiji (ImageJ). Significant differences were found between the Control and the I/R groups (219.2 ± 12.64 vs. 52.07 ± 11.87 total nuclei/mm^2^, *p* ≤ 0.05), between the I/R and the PostC groups (52.07 ± 11.87 vs. 230.5 ± 55.3 total nuclei/mm^2^, *p* ≤ 0.05) and among the PostC and the PostC + Lat A groups (230.5 ± 55.3 vs. 52.5 ± 6.71 total nuclei/mm^2^, *p* ≤ 0.05) (Supplementary Figure S2).

Moreover, electronic microscopy images showed uniformly arranged myocardial fibers, along with clear muscle segments and preserved mitochondria in the Control and PostC groups, whereas in the I/R group and the Post + Lat A groups, edema between muscle fibers and mild fragmentation, along with mitochondrial swelling and even partial disappearance of such organelles, were observed (Figure 2b).

**Figure 3:**
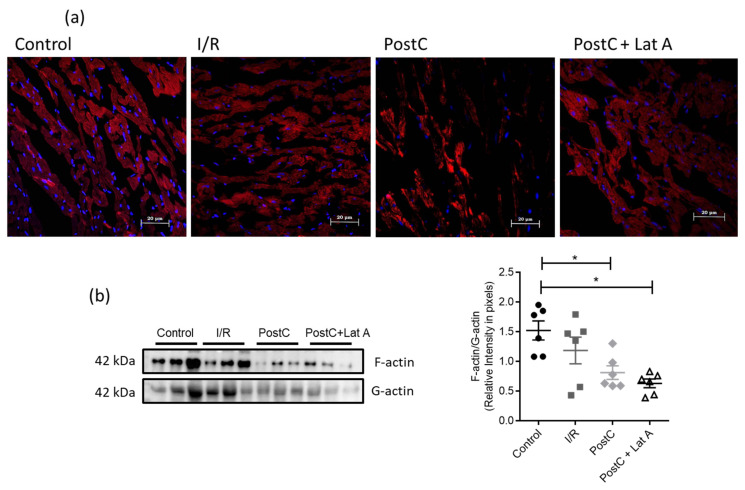
Changes in F-actin staining in post-conditioned hearts treated with latrunculin A. (**a**) Representative fluorescence microscopy images of rat heart sections from the different experimental groups. Cell nuclei were counterstained with DAPI and F-actin with phalloidin-iFluor 647. (**b**) F-actin and G-actin content in fractions obtained from cardiac tissue. Homogenates of each heart were lysed in F-actin stabilizing buffer and G-actin fractions were collected after high-speed centrifugation. Then, the pellets containing the F-actin fraction were depolymerized and subjected along with G-actin fractions to SDS-PAGE and immunoblotting with anti-β-actin antibody. Densitometric analysis of the blot was carried out using ImageJ software and the statistical significance of the data was assessed by a one-way ANOVA test. *p*-value for * is <0.05. Bars represent the mean ± SEM, of six independent preparations.

Phalloidin is a bicyclic heptapeptide from poisonous mushrooms, that binds to actin filaments more tightly than to actin monomers, therefore we used this compound to evaluate the possible changes in the F-actin microfilament cytoskeleton in the PostC + Lat A group. F-actin staining with phalloidin slightly diminished in I/R hearts, as compared to the Control hearts; whereas F-actin staining was reduced in the PostC + Lat A group, as compared with both the Control and PostC hearts (Figure 3a); however, when measuring the F-actin/G-actin, no differences were found between the PostC and the PostC + Lat A groups (Figure 3b).

**Figure 4:**
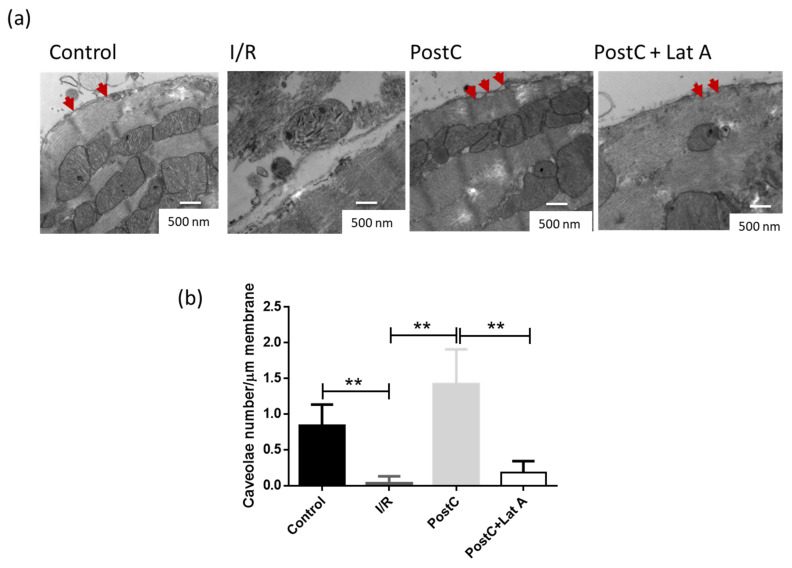
Latrunculin A diminishes the caveolae content in post-conditioned hearts. (**a**) Representative electron microscopy images showing the presence of caveolae in the sarcolemma of the different experimental groups (red arrows). (**b**) Quantitative analysis of the observed caveolae along the sarcolemma from all groups. The statistical significance of the data was assessed by a one-way ANOVA test. *p*-value for ** is <0.001. Values are the mean ± SEM, n = 20 micrographs per group.

Moreover, the structural analysis revealed characteristic flask-shaped invaginations in the sarcolemma or internalized vesicles near its boundary, identified as caveolae. Such structures diminished in I/R hearts, as compared with both the Control and PostC groups (*p* < 0.05). The addition of the cytoskeleton disruptor latrunculin A reduced the caveolae number in the PostC hearts, although the structures remained more abundant than in the I/R group (Figure 4). We also observed that an increased caveolae abundance correlates with preserved sarcolemmal mitochondria in both the Control and PostC hearts.

### 3.2. Cav-1 and Cav-3 Co-Localization with Actin in Post-Conditioned Hearts Treated with Latrunculin A

**Figure 5:**
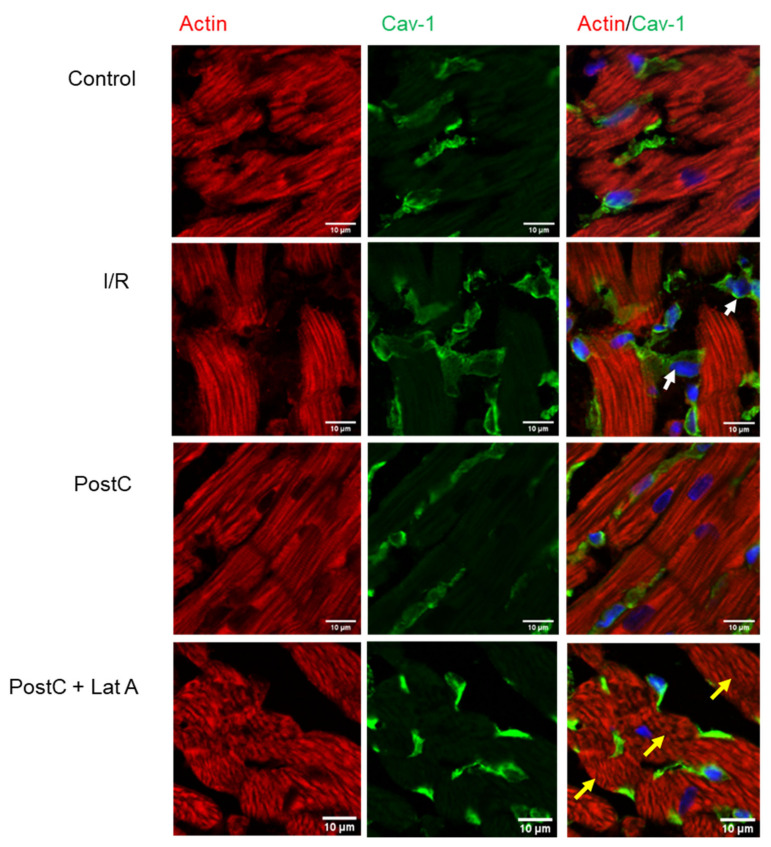
Co-localization of caveolin-1 and actin in post-conditioned hearts treated with latrunculin A. Representative confocal laser microscopy of the sections of frozen heart tissue after double-immunofluorescence staining of caveolin-1 (green) and actin (red). Cell nuclei were counterstained with DAPI.

Co-localization of cav-1 and total actin was first evaluated, as it has been reported to be expressed in cardiac muscle cells as well as in other cell types, such as endothelial cells, fibroblasts and adipocytes. Cav-1 was not detectable in muscle cells, having a major expression in the endothelium and fibroblasts (white arrows). Moreover, total actin staining showed well-organized heart fibers in both the Control and PostC hearts, whereas I/R hearts showed disorganized fibers and edema. We observed that cardiomyocytes from PostC + Lat A hearts had an unusual structure, as even transverse fibers presented a feathery feature (yellow arrows), possibly resulting from the actin filament disruption (Figure 5).

**Figure 6:**
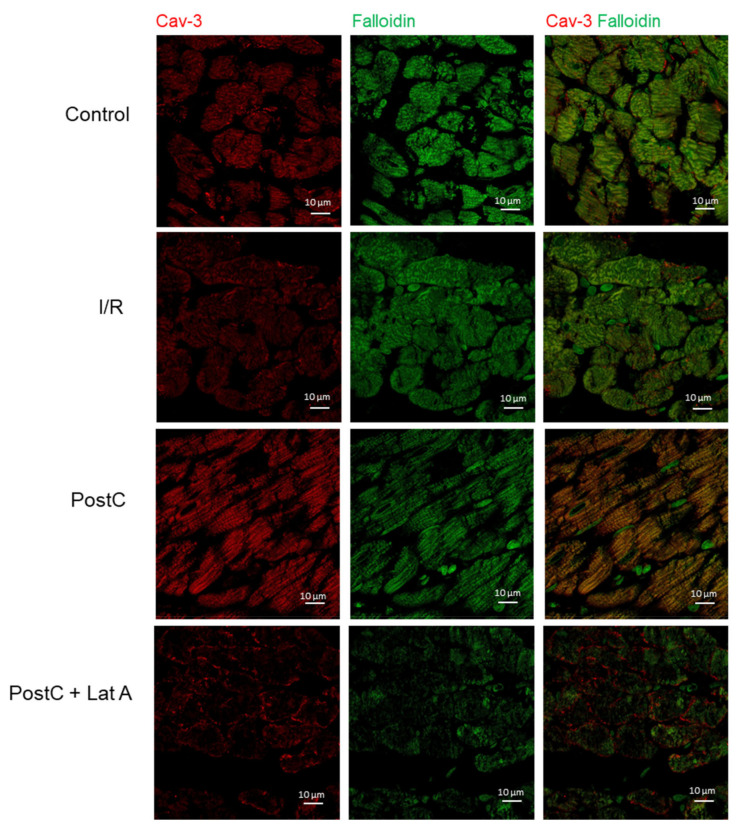
Co-localization of caveolin-3 and F-actin in post-conditioned hearts treated with latrunculin A. Samples were labeled with primary antibodies, as indicated on micrographs: cav-3 (red), phalloidin (green) color. Prior to merging the confocal images, signals for red and green fluorescence were adjusted to comparable levels. The yellow color indicates co-localization of two labeled antigens for cav-3 and F-actin. Confocal laser scanning images were obtained at 63× magnification.

Moreover, the isoform cav-3 was located in the sarcolemma of all groups, although we observed that the signal diminished in the I/R group. Phalloidin staining was similar in the Control, I/R and PostC groups, but diminished notably in the PostC + Lat A hearts. Remarkably, in the PostC cardiac muscle cells, cav-3 and phalloidin were labeled at the same loci that might include T-tubules staining. Such a signal was lost in the PostC + Lat A, along with augmented fluorescence of cav-3 in the sarcolemma (Figure 6).

### 3.3. Latrunculin A Reduces the Actin Association with Detergent-Insoluble Caveolae in PostC Hearts

**Figure 7:**
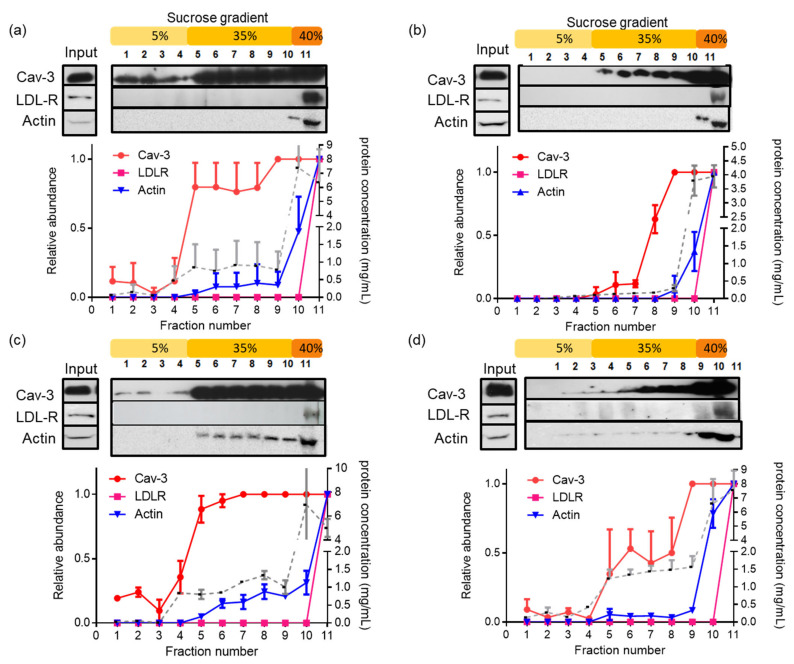
Effect of latrunculin A on the cav-3 and actin distribution in lipid rafts microdomains obtained from post-conditioned hearts. Sucrose gradient fractions obtained after TX100 solubilization of homogenates from the Control (**a**); I/R (**b**); PostC (**c**) and PostC + Lat A (**d**) hearts were analyzed by western blot using anti-cav-3, anti-actin mAb and anti-LDL-R. It is shown that the total protein content in each fraction (dotted line) and the relative abundance of the evaluated proteins (■) LDL-R (110 kDa); (●) cav-3 (17 kDa) and (▼) actin (42 kDa). Results represent the mean ± SEM from three independent experiments.

Caveolae are specialized sub-types of lipid rafts, formed after the segregation of glycosphingolipids and cholesterol within the exoplasmic leaflet of the plasmatic membrane, characterized as “liquid-ordered” domains resistant to solubilization with nonionic detergents at a low temperature [21]. To determine if caveolae diminution in PostC hearts in the presence of latrunculin A was associated with loss of the association between cav-3 and actin, as observed in Figure 6, we isolated the low-buoyant density fractions from detergent-insoluble membranes from heart tissue. PostC hearts showed a characteristic low-buoyant density band around the 5–35% interphase of the gradient, which was less visible in the other groups (Supplementary Figure S3). The content of cav-3, actin, and LDL-R (a non-lipid raft protein) was determined along the gradient by the western blot analysis using specific antibodies (Figure 7). Cav-3 migrated towards the top of the gradient in the Control, PostC (Figure 7a,c) and to a lesser extent, in the Post +Lat A groups (Figure 7d), demonstrating the presence of caveolae in the low-density membranes, in concordance with the caveolae number, increase, as observed by electronic microscopy. Moreover, the cav-3 content was lower along the gradient in the I/R group (Figure 7b). Although actin was not detected along the sucrose gradient neither in the Control or I/R groups, the actin distribution resembled the mobility of the detergent-insoluble cav-3 in the PostC hearts (Figure 7c), which was clearly diminished in the PostC + Lat A group (Figure 7d), supporting a possible link between caveolae/lipid rafts and the cytoskeleton in post-conditioned hearts. The LDL receptor (LDL-R) was used as a negative control, as it does not form part of the caveolar structure. As expected, LDL-R remained at the bottom of the gradient in all groups.

**Figure 8:**
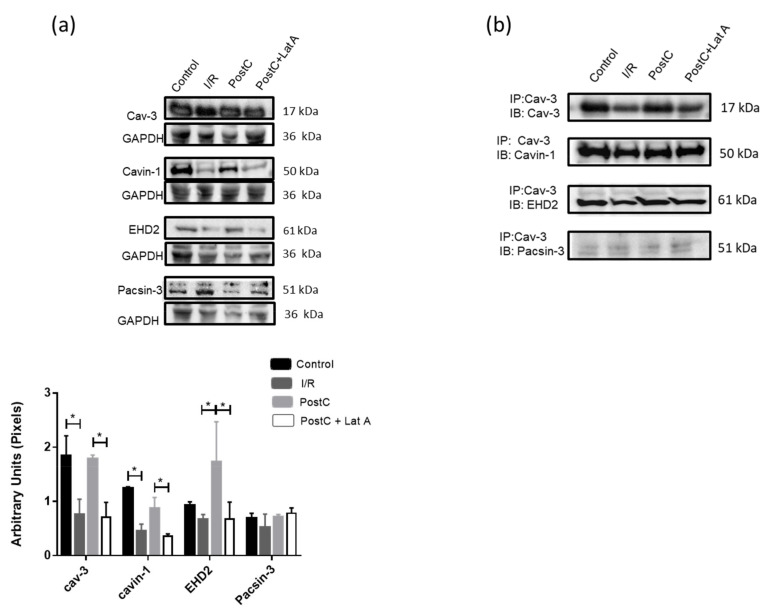
Latrunculin A modifies the content of the caveolar proteins in post-conditioned hearts. (**a**) Representative western blot images of cav-3, cavin-1, EHD2, and pacsin-3 in cardiac homogenates from the different experimental groups. GAPDH was used as a loading control. Densitometric analysis of cav-3, cavin-1, EHD2 and pacsin-3 content normalized with GADPH. The statistical significance of the data was assessed by a two-way ANOVA test, followed by Tukey’s multiple comparisons test. *p*-value for * is <0.05. Values presented in the graph are the mean ± SEM, of three independent samples. (**b**) Immunoprecipitation (IP)-western blotting (IB) analysis shows associations of cav-3, cavin-1, EHD2, and pacsin-3 in homogenates from the experimental groups. n = 2.

We also evaluated cavin-1 (PTRF), a major reported caveolae protein, along with other putative components of these structures: EHD-2 and pacsin-3. Cavin-1 (PTRF) was concentrated in the bulk cellular proteins in all groups, although a small fraction was also distributed along the 35% layer in the PostC hearts (Supplementary Figure S4). Neither EHD-2 nor pacsin-3 moved to the low-density layers of the sucrose gradients (Supplementary Figure S5), even though both proteins were detected in the homogenates from all groups (Figure 8a). Actin and cav-3 localization in the lipid raft fraction supports the interaction of caveolae and the cytoskeleton in cardioprotective conditions.

### 3.4. Changes in Caveolar Proteins in PostC Hearts Treated with Latrunculin A

In Figure 8a, it is also observed that the cav-3 and cavin-1 content were reduced in the I/R group, as compared with the Control; and that the recovery of such proteins along with the increase of EHD2 in the PostC hearts was inhibited by the latrunculin A treatment. We would like to remark that, to our knowledge, this is the first time that EHD2 content increase is associated with cardio-protection and its reduction with the actin cytoskeleton disruption.

Moreover, no changes were detected in pacsin-3 levels among the groups (Figure 8a). It is worth mentioning that the observed molecular weight of this protein was higher than that reported (48–51 kDa). Therefore, we made comparisons on the mobility of the protein with homogenates from the liver and kidney. A band of 51 kDa was only detected in the liver, whereas in the kidney and in heart homogenates, the main band was around 60 kDa (Supplementary Figure S6).

Next, we performed co-immunoprecipitation protocols, to determine whether changes in protein-protein interactions between caveolae forming proteins might result from the actin cytoskeleton disruption. As shown in Figure 8b, cav-3 co-immunoprecipitates with cavin-1 (PTRF), EHD2, and to a much smaller extent with pacsin-3 in all groups, disruption of the cytoskeleton in the PostC + Lat A groups did not lower the interaction between cav-3 and cavin-1 or EHD2. These results suggest that the interactions between the caveolar proteins are maintained independently of the actin cytoskeleton association with the caveolae.

### 3.5. Actin-Cytoskeleton Disruption Reduces the Cholesterol Delivery towards the Mitochondria and Impacts Their Function

**Figure 9:**
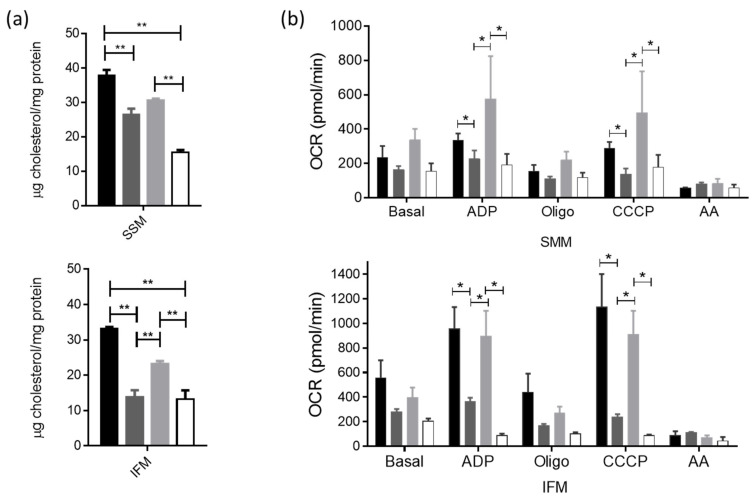
Cholesterol content and oxygen consumption rate in mitochondrial subpopulations from post-conditioned hearts treated with latrunculin A. (**a**) Cholesterol content in subsarcolemmal mitochondria (SMM) and interfibrillar mitochondria (IFM). The statistical significance of the data was assessed by a one-way ANOVA test. *p*-value for ** is <0.001. (**b**) Oxygen consumption rate in SSM and IFM. Statistical significance of the data was assessed by a two-way ANOVA test. *p*-value for * is < 0.05. Values are the mean ± SEM of at least three different mitochondrial preparations.

In addition to being critical to caveolae formation, cholesterol is required for the caveolin incorporation into the lipid raft domains. Accordingly, we determined that the changes in mitochondrial cholesterol levels were a complementary parameter to sustain caveolae interaction with such organelles. We obtained preparations of both subsarcolemmal (SSM) and interfibrillar (IFM) mitochondria to determine the effect of actin-cytoskeleton disruption on the function of these organelles at their different distributions. We observed that cholesterol was reduced in both mitochondrial subpopulations obtained from the I/R hearts, as compared with those from the Control group. Post-conditioning augmented the cholesterol levels only in IFM, whereas both populations in the PostC + Lat A groups showed lower cholesterol content than in the PostC mitochondria (Figure 9a). Next, we measured oxygen consumption rates (OCRs) at basal, phosphorylating, and uncoupled states in isolated mitochondria from all groups, to correlate the function of these organelles with the observed cholesterol changes after latrunculin A treatment (Figure 9b). Basal respiration (State 2) and non-ADP-stimulated respiration (State 4o) rates of both SMM and IFM were comparable between all groups. ADP-stimulation respiration (State 3), coupled with ATP synthesis diminished in both SSM and IFM from the I/R hearts, as compared to those obtained from the Control hearts. In PostC, this parameter was recovered in both mitochondrial subpopulations, whereas such an increase was abolished in the presence of latrunculin A, indicating a loss of mitochondrial oxidative phosphorylation capacity. Furthermore, the CCCP-stimulated respiration (State 3u) or maximal respiratory capacity increased in the mitochondria from PostC hearts, in comparison with the I/R group, a condition that was reverted by the latrunculin A action. Taken together, our results demonstrate that mitochondria from hearts treated with the cytoskeleton disruptor, lose their electron transport capacity in association with the cardio-protective effect of post-conditioning driven by caveolae.

## 4. Discussion

Latrunculins have been used to study the involvement of actin in several cellular processes. It was reported that latrunculin A induces the loss of actin-based stabilizing scaffolds that regulate the formation and maintenance of cardiomyocyte plasma membrane subdomains, such as caveolae [22]. Accordingly, the data presented in this study provide evidence that the actin-depolymerizing agent latrunculin A, abolishes the cardioprotective effect of post-conditioning, modifying the plasma membrane ultrastructure, diminishing the caveolae formation, and impacting the mitochondrial function.

Even though caveolae are considered as relatively immobile structures, they are internalized and establish trafficking routes either by the classic endocytic pathway or toward intracellular organelles [23]. The proposal that the cytoskeleton might facilitate caveolar subcellular signaling, it is sustained by the different observations. For example, caveolin-3 shifts from the cytoskeleton and TX100 insoluble fractions to detergent soluble fractions in ventricular myocytes treated with the cholesterol-removing agent MβCD [24]. Furthermore, the simian virus 40 (SV40) invades host cells through caveolae, after transient changes in the actin cytoskeleton [25] and cytoskeleton disorganization is associated with the reduction of caveolin phosphorylation, along with adenylate cyclase pathway inactivation in cardiomyocytes [15].

Once we determined that the actin-cytoskeleton disruption abolishes the cardioprotective effect of post-conditioning and reduces the caveolae number, we evaluated whether cav-3 associates with actin-cytoskeleton, by measuring the co-localization of both proteins and analyzed their distribution in “lipid rafts” fractions. The immunofluorescence images show a clear co-localization of cav-3 with F-actin in post-conditioned cardiac tissue, but not in the other groups. Accordingly, we observed that cav-3 and total actin co-fractionated in low-density fractions from post-conditioned heart homogenates, and that such an association was decreased in the presence of the actin-depolymerization compound. These findings correlate with reports in which the knockdown of cavin-3 or treatment with latrunculin A changed the distribution of the signaling proteins MEK and ERK into high-sucrose density fractions [26]. In this regard, it has been reported that proteins associated to actin, have binding sites to caveolae conforming proteins [4]. Specifically, cavin-3 with the participation of myosin-1c anchors caveolae to peripheral actin [27], whereas pacsin interacts directly with actin [16]. There is also evidence of a cavin-1 association with the cytoskeleton, as the silencing of this protein promotes the actin diminution in “lipid rafts” obtained from adipocytes [26], such reports reinforce our results, overall supporting the hypothesis of the association between caveolae and cytoskeleton.

We also evaluated the effect of the actin-cytoskeleton disruption on the content of caveolae forming proteins, cav-3, cavin-1, EHD2 and pacsin-3. Cav-3, and cavin-1 are considered the minimal caveolae coat machinery necessary to form invaginated caveolae [28] in a process in which the membrane interacting with cavins and caveolins is inserted into membranes enriched with cholesterol, and phospholipids favor the formation of such structures [29]. Conversely, the ATPase EHD2, is more related to caveolae stabilization [30]. This protein is a member of the dynamin superfamily of GTPases, which oligomerizes into the lipid interphase forming ring-like assemblies at the neck of the caveolae [31]. Accordingly, with the caveolae number decrease, cav-3, cavin-1 and EHD2 content diminishes in the PostC + Lat A hearts, suggesting that the actin-cytoskeleton participates in the caveolae formation. Conversely, pacsin-3 (also called syndapin III) a muscle-enriched isoform of the syndapin family of F-BAR proteins that binds to EHD2 and that has been suggested to contribute to stabilizing the caveolae neck [32], does not change in the PostC + Lat A heart homogenates. The association between cav-3, cavin-1, EHD2 and pacsin-3 was confirmed by immunoprecipitation. Only cav-3 and to some extent cavin-1, resembled the differences observed in homogenates from the different groups (Figure 8) and with caveolae number (Figure 4), in spite that pacsin-3 has been described to contain actin-binding domains [33]. Remarkably, cav-3 co-immunoprecipitated with pacsin-3 to a much more minor extent than with cavin-1 and EHD2, confirming the suggestion that this protein may not represent a general and integral part of cav-3 coats [32]. A possible explanation might be that EDH2 and pacsin have additional functions outside of the caveolae. It has been proposed that pacsin 2 is required only at one step in which caveolin enriched microdomains conform the caveolae, but contrary to the cavin proteins, pacsin does not associate constitutively to the invaginated structure [34].

Caveolae-driven cardioprotective signaling linked to the maintenance of the mitochondrial function, has been established by different groups, [12,35,36]. Such evidence sustains that caveolae from the plasmatic membrane are critical to connecting the extracellular milieu and intracellular signaling to regulate energy and mitochondrial metabolism [37]. To our knowledge, there are no reports that the cardio-protective signaling transmission to mitochondria relies on the association between the caveolae and actin-cytoskeleton. However, there are studies supporting the possible interrelationship between such subcellular structures. Examples are the demonstration that the myosin II-actin interaction induces cardiomyocyte apoptosis and mitochondrial fission in I/R [38]; that mutations in actin are related to intrinsic apoptosis [39], and the proposal that in δ-opioid-induced cardio-protection, caveolin translocation to mitochondria might be related with the cytoskeleton or specific chaperone proteins [36].

As described above, it is tempting to speculate that the cytoskeleton might play a relevant role in the crosstalk between caveolae and mitochondria under the cardio-protective signaling activated by post-conditioning. We are aware that even contractile dysfunction is a reliable measure of ischemic injury and it is not necessarily synonymous of cell death. We do not discard that other levels of regulation besides disruption of actin-skeleton, and reduction of the structural/functional interaction between sarcolemmal caveolae and mitochondria in post-conditioning cardio-protection, might be exerted by latrunculin A.

## 5. Conclusions

Communication between caveolae containing cardioprotective signaling and mitochondria is related to the actin-cytoskeleton, promoting cardiac resistance to ischemic reperfusion damage.

## Data Availability

Data are contained within the article and Supplementary Materials.

## Supplementary Materials

The following supporting information can be downloaded at: https://www.mdpi.com/article/10.3390/cells12030492/s1, Figure S1: Effect of latrunculin A incubation in heart rate and LVDP in the Control and PostC hearts; Figure S2: Nuclear quantification of H&E-stained tissue; Figure S3: Lipid raft extraction with TX100 and isolation in discontinuous sucrose gradient; Figure S4: Cavin-1 (PTRF) detection in the lipid rafts extracted with TX100 and isolated in discontinuous sucrose gradients; Figure S5: EDH2 and pacsin-3 detection in the lipid rafts extracted with TX100 and isolated in discontinuous sucrose gradients, Figure S6: Pacsin-3 detection in the homogenates from the kidney, heart and liver.

## References

[1] Shaul P.W., Anderson R.G. (1998). Role of plasmalemmal caveolae in signal transduction. Am. J. Physiol..

[2] Song K.S., Scherer P.E., Tang Z., Okamoto T., Li S., Chafel M., Chu C., Kohtz D.S., Lisanti M.P. (1996). Expression of caveolin-3 in skeletal, cardiac, and smooth muscle cells. Caveolin-3 is a component of the sarcolemma and co-fractionates with dystrophin and dystrophin-associated glycoproteins. J. Biol. Chem..

[3] Feron O., Balligand J.L. (2006). Caveolins and the regulation of endothelial nitric oxide synthase in the heart. Cardiovasc. Res..

[4] Stahlhut M., van Deurs B. (2000). Identification of filamin as a novel ligand for caveolin-1: Evidence for the organization of caveolin-1-associated membrane domains by the actin cytoskeleton. Mol. Biol. Cell.

[5] Heusch G. (2015). Molecular basis of cardioprotection: Signal transduction in ischemic pre-, post-, and remote conditioning. Circ. Res..

[6] Davidson S.M., Hausenloy D., Duchen M.R., Yellon D.M. (2006). Signalling via the reperfusion injury signalling kinase (RISK) pathway links closure of the mitochondrial permeability transition pore to cardioprotection. Int. J. Biochem. Cell Biol..

[7] Tsibulnikov S.Y., Maslov L.N., Naryzhnaya N.V., Ma H., Lishmanov Y.B., Oeltgen P.R., Garlid K. (2018). Role of protein kinase C, PI3 kinase, tyrosine kinases, NO-synthase, KATP channels and MPT pore in the signaling pathway of the cardioprotective effect of chronic continuous hypoxia. Gen. Physiol. Biophys..

[8] Quinlan C.L., Costa A.D., Costa C.L., Pierre S.V., Dos Santos P., Garlid K.D. (2008). Conditioning the heart induces formation of signalosomes that interact with mitochondria to open mitoKATP channels. Am. J. Physiol. Heart Circ. Physiol..

[9] Hernández-Reséndiz S., Zazueta C. (2014). PHO-ERK1/2 interaction with mitochondria regulates the permeability transition pore in cardioprotective signaling. Life Sci..

[10] Lupi Herrera E., Gaspar J., González-Pacheco H., Martínez-Sánchez C., Pastelín-Hernández G., Luna-Ortiz P., Chávez-Cosio E. (2006). Reperfusion and postconditioning in acute ST segment elevation myocardial infarction. A new paradigm for the treatment of acute myocardial infarction. From bench to bedside?. Arch. Cardiol. Mex..

[11] Sun J., Nguyen T., Aponte A.M., Menazza S., Kohr M.J., Roth D.M., Patel H.H., Murphy E., Steenbergen C. (2015). Ischaemic preconditioning preferentially increases protein S-nitrosylation in subsarcolemmal. Cardiovasc. Res..

[12] García-Niño W.R., Correa F., Rodríguez-Barrena J.I., León-Contreras J.C., Buelna-Chontal M., Soria-Castro E., Hernández-Pando R., Pedraza-Chaverri J., Zazueta C. (2017). Cardioprotective kinase signaling to subsarcolemmal and interfibrillar mitochondria is mediated by caveolar structures. Basic Res. Cardiol..

[13] Revenu C., Athman R., Robine S., Louvard D. (2004). The co-workers of actin filaments: From cell structures to signals. Nat. Rev. Mol. Cell Biol..

[14] Jasper J.R., Post S.R., Desai K.H., Insel P.A., Bernstein D. (1995). Colchicine and cytochalasin B enhance cyclic AMP accumulation via postreceptor actions. J. Pharmacol. Exp. Ther..

[15] Head B.P., Patel H.H., Roth D.M., Murray F., Swaney J.S., Niesman I.R., Farquhar M.G., Insel P.A. (2006). Microtubules and actin microfilaments regulate lipid raft/caveolae localization of adenylyl cyclase signaling components. J. Biol. Chem..

[16] Kostan J., Salzer U., Orlova A., Törö I., Hodnik V., Senju Y., Zou J., Schreiner C., Steiner J., Meriläinen J. (2014). Direct interaction of actin filaments with F-BAR protein pacsin2. EMBO Rep..

[17] Hernández-Reséndiz S., Roldán F.J., Correa F., Martínez-Abundis E., Osorio-Valencia G., Ruíz-de-Jesús O., Alexánderson-Rosas E., Vigueras R.M., Franco M., Zazueta C. (2013). Postconditioning protects against reperfusion injury in hypertensive dilated cardiomyopathy by activating MEK/ERK1/2 signaling. J. Card. Fail..

[18] Smyth J.W., Vogan J.M., Buch P.J., Zhang S., Fong T.S., Hong T., Shaw R.M. (2012). Actin cytoskeleton rest stops regulate anterograde traffic of connexin 43 vesicles to the plasma membrane. Circ. Res..

[19] Lowry O.H., Rosebrough N.J., Farr A.L., Randall R.J. (1951). Protein measurement with the Fohn phenol reagent. J. Biol. Chem..

[20] Rudel L.L., Morris M.D. (1973). Determination of cholesterol using o-phthalaldehyde. J. Lipid Res..

[21] Sezgin E., Levental I., Mayor S., Eggeling C. (2017). The mystery of membrane organization: Composition, regulation and roles of lipid rafts. Nat. Rev. Mol. Cell Biol..

[22] Itoh T., Erdmann K.S., Roux A., Habermann B., Werner H., De Camilli P. (2005). Dynamin and the actin cytoskeleton cooperatively regulate plasma membrane invagination by BAR and F-BAR proteins. Dev. Cell.

[23] McMahon K.A., Zhu M., Kwon S.W., Liu P., Zhao Y., Anderson R.G.W. (2006). Detergent-free caveolae proteome suggests an interaction with ER and mitochondria. Proteomics.

[24] Haque M.Z., McIntosh V.J., Abou-Samra A.B., Mohammad R.M., Lasley R.D. (2016). Cholesterol Depletion Alters Cardiomyocyte Subcellular Signaling and Increases Contractility. PLoS ONE.

[25] Pelkmans L., Püntener D., Helenius A. (2002). Local actin polymerization and dynamin recruitment in SV40-induced internalization of caveolae. Science.

[26] Liu L., Pilch P.F. (2008). A critical role of cavin (polymerase I and transcript release factor) in caveolae formation and organization. J. Biol. Chem..

[27] Hernandez V.J., Weng J., Ly P., Pompey S., Dong H., Mishra L., Schwarz M., Anderson R.G.W., Michaely P. (2013). Cavin-3 dictates the balance between ERK and Akt signaling. ELife.

[28] Hubert M., Larsson E., Lundmark R. (2020). Keeping in touch with the membrane; protein- and lipid-mediated confinement of caveolae to the cell surface. Biochem. Soc. Trans..

[29] Parton R.G., Tillu V., McMahon K.A., Collins B.M. (2021). Key phases in the formation of caveolae. Curr. Opin. Cell Biol..

[30] Yeow I., Howard G., Chadwick J., Mendoza-Topaz C., Hansen C.G., Nichols B.J., Shvets E. (2017). EHD proteins cooperate to generate caveolar clusters and to maintain caveolae during repeated mechanical stress. Curr. Biol..

[31] Daumier O., Lundmark R., Vallis Y., Martens S., Butler P.J.G., McMahon H.T. (2007). Architectural and mechanistic insights into an EHD ATPase involved in membrane remodelling. Nature.

[32] Seemann E., Sun M., Krueger S., Tröger J., Hou W., Haag N., Schüler S., Westermann M., Huebner C.A., Romeike B. (2017). Deciphering caveolar functions by syndapin III KO-mediated impairment of caveolar invagination. Elife.

[33] Qualmann B., Kelly R.B. (2000). Syndapin isoforms participate in receptor-mediated endocytosis and actin organization. J. Cell Biol..

[34] Hansen C.G., Howard G., Nichols B.J. (2011). Pacsin 2 is recruited to caveolae and functions in caveolar biogenesis. J. Cell Sci..

[35] Fridolfsson H.N., Roth D.M., Insel P.A., Patel H.H. (2014). Regulation of intracellular signaling and function by caveolin. FASEB J..

[36] Wang J.W., Xue Z.Y., Wu A.S. (2019). Mechanistic insights into δ-opioid-induced cardioprotection: Involvement of caveolin translocation to the mitochondria. Life Sci..

[37] Kuznetsov A.V., Javadov S., Grimm M., Margreiter R., Ausserlechner M.J., Hagenbuchner J. (2020). Crosstalk between Mitochondria and Cytoskeleton in Cardiac Cells. Cells.

[38] Li F., Fan X., Zhang Y., Zhang Y., Ma X., Kou J., Yu B. (2018). Inhibition of myosin IIA-actin interaction prevents ischemia/reperfusion induced cardiomyocytes apoptosis through modulating PINK1/Parkin pathway and mitochondrial fission. Int. J. Cardiol..

[39] Boldogh I.R., Pon L.A. (2006). Interactions of mitochondria with the actin cytoskeleton. Biochim. Biophys. Acta.

