# Supplementary material for: Actin-Cytoskeleton Drives Caveolae Signaling to Mitochondria during Postconditioning
Source: Cells. 2023 Feb 2;12(3):492. doi: 10.3390/cells12030492 (PMC9914812; doi:10.3390/cells12030492)
Supplement: Supplementary file 1 [file cells-12-00492-s001.zip › cells-2095196-supplementary.pdf]

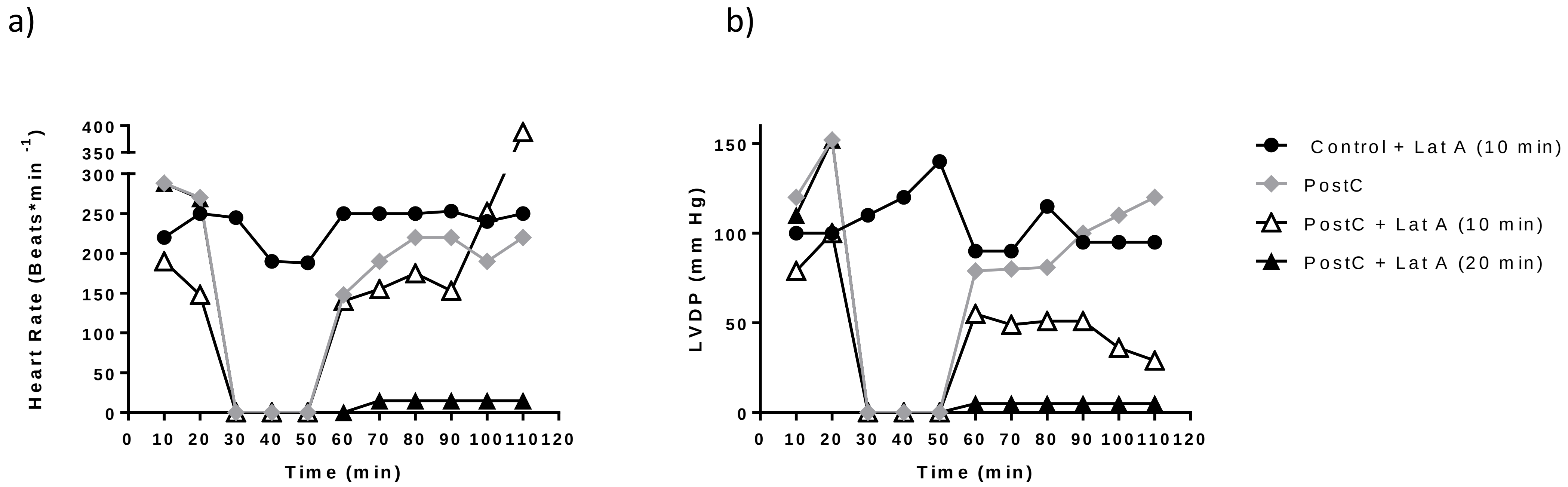

Supplementary Figure S1. Effect of Latrunculin A incubation in a) Heart rate and b) LVDP in Control and PostC hearts. Hearts were perfused with Latrunculin A (1 µM) during the stabilization period before ischemia. Data represents the mean of 2 different preparations of the indicated groups.

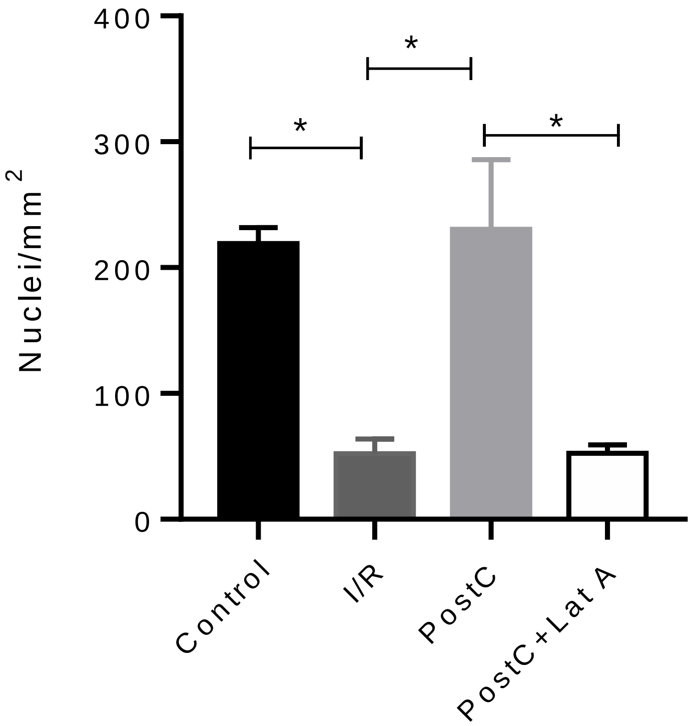

Supplementary Figure S2. Nuclear quantification of H&E-stained tissues with IHC Plugin Fiji (ImageJ) and reported as total nuclei per area. Histological analysis was performed in 4-5 sections per left ventricle of different preparations and evaluated in a blinded manner. Results are media ± SEM. **p ≤ 0.05*

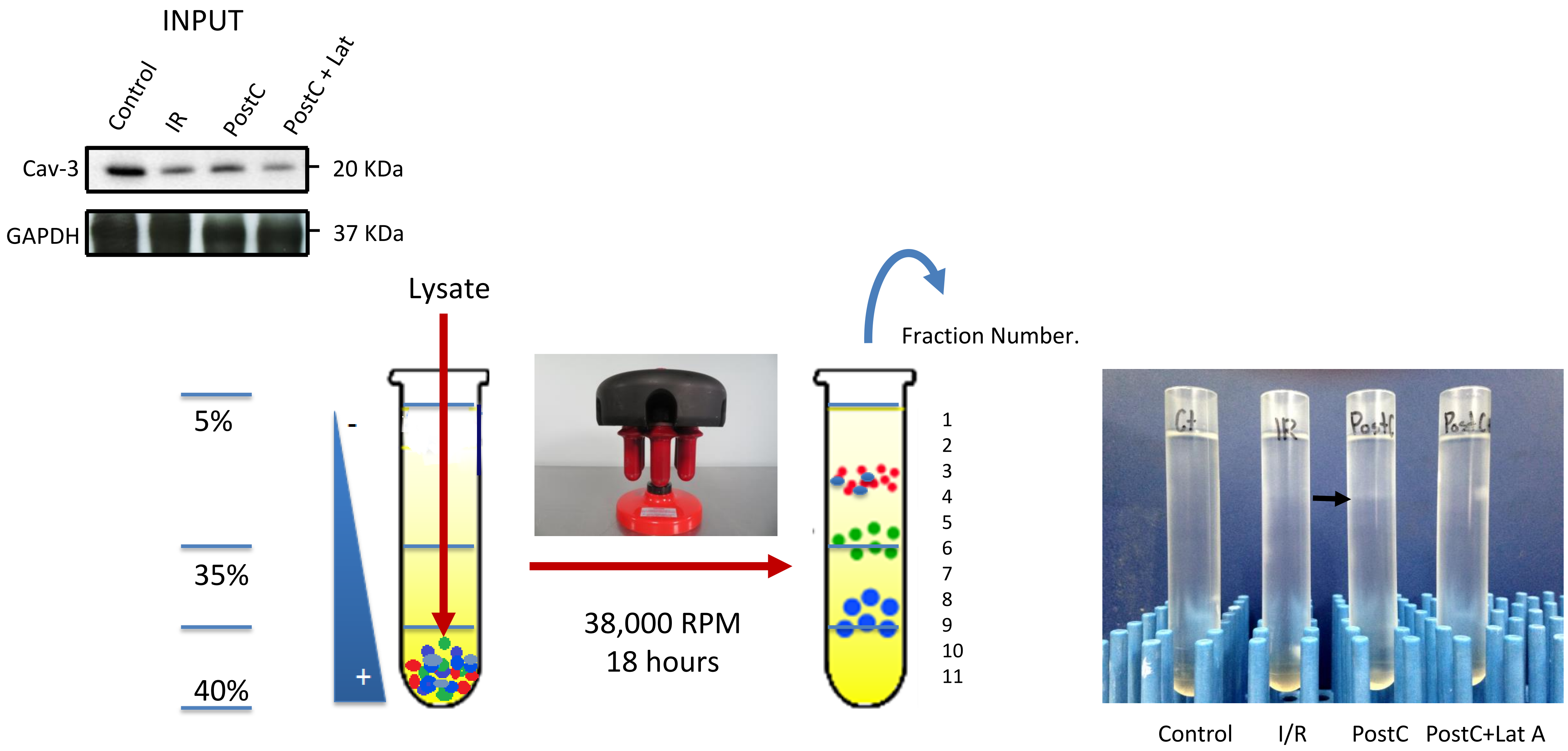

Supplementary Figure S3. Lipid raft extraction with TX100 and isolation in discontinuous sucrose gradients. Representative western blot image showing cav-3 and GAPDH input (left) and image of low-buoyant density band in the sucrose gradient (fractions number 4-5). Total protein was determined and cav-3, actin and LDL-R content was analyzed.

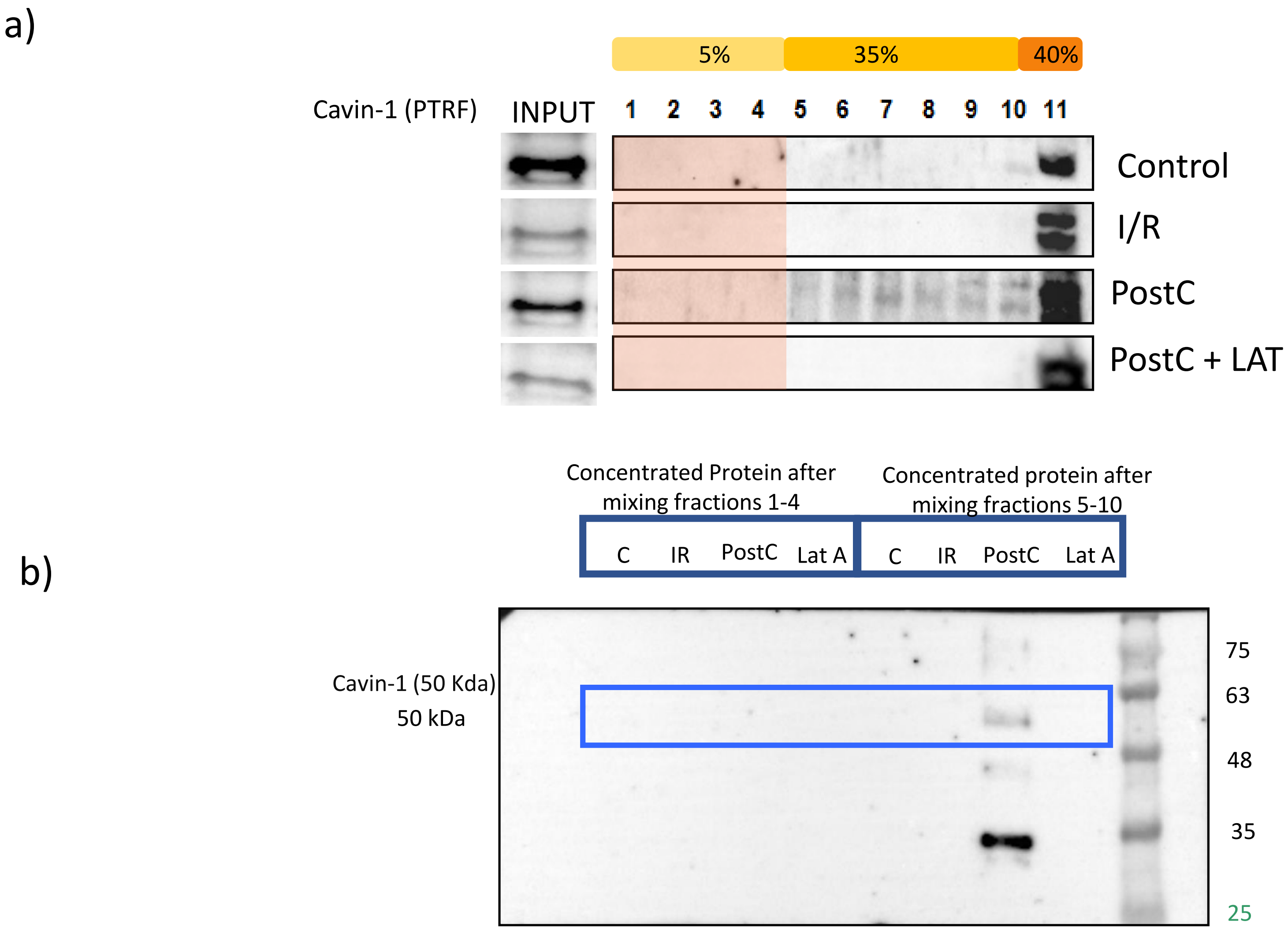

Supplementary Figure S4. Cavin-1 (PTRF) detection in lipid rafts extracted with TX100 and isolated in discontinuous sucrose gradients.
a) Representative western blot images showing cavin-1 input and protein mobility along the gradient in Control, I/R, PostC and PostC + Lat A groups.
b) Western blot image of pooled samples from fractions 1-4 and 5-10.

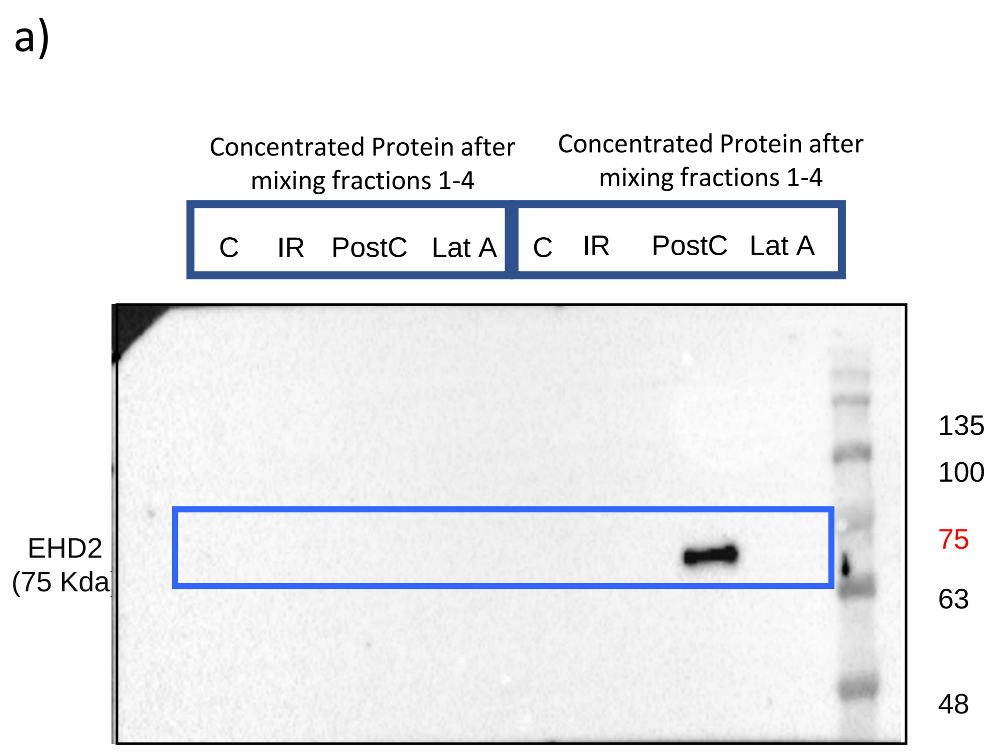

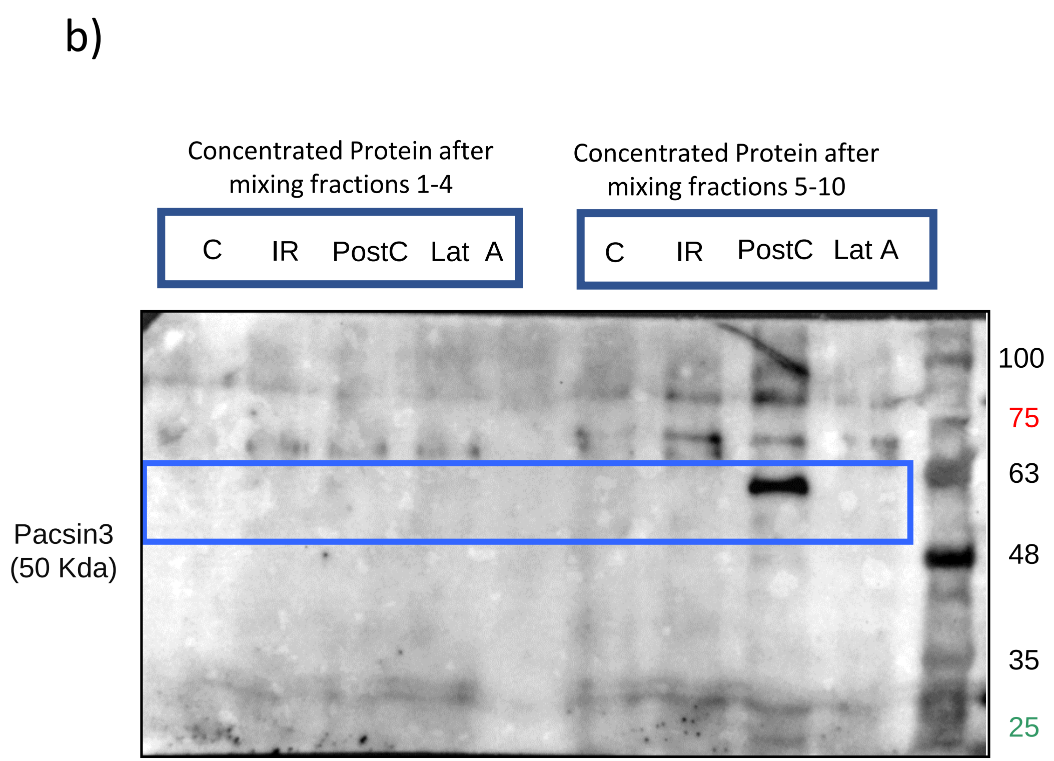

Supplementary Figure S5. EHD2 AND Pacsin 3detection in lipid rafts extracted with TX100 and isolated in discontinuous sucrose gradients.

A) Representative western blot images showing EHD2 and Pacsin 3 content in pooled samples from fractions 1-4 and 5-10 of the sucrose gradient. In PostC samples it was detected a strong molecular band, of higher weight than that reported for pacsin-3.

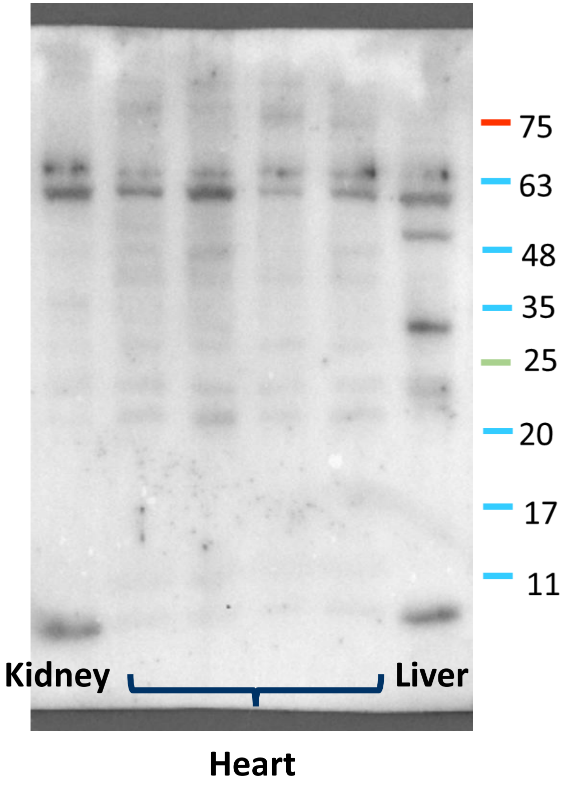

Supplementary Figure S6. Pacsin-3 detection in homogenates from kidney, heart and liver. A band of the predicted pacsin-3 molecular weight (51 kDa, was detected in liver homogenates along with bands resolved above this marker. As these signals were present in homogenates from kidney, heart and liver we present such image, as pacsin-3 content.
